# The implications of inefficient markets for executive pay comparison: The case of China and Poland

**DOI:** 10.3389/fpsyg.2022.888977

**Published:** 2022-11-21

**Authors:** Yanqiu Liu, Hanmin Liu

**Affiliations:** ^1^School of Business Administration, Southwestern University of Finance and Economics, Chengdu, China; ^2^School of Management, Jinan University, Guangzhou, China

**Keywords:** corporate governance, executive compensation, pay comparison, comparative research, emerging market

## Abstract

Although there is a large volume of literature on executive compensation, few of these studies have focused on executive pay comparisons and even fewer on the antecedents of executive pay comparisons. This paper fills this gap in executive pay comparison literature by beginning with executive pay comparison behaviors, and then the elements that influence executive pay comparison behaviors are discussed. A questionnaire survey found that executive pay comparison behaviors exist in both China and Poland. Furthermore, the findings show that the factors influencing executive pay comparison behaviors are different in the two countries. In China, there is a significant relationship between executive pay comparison behaviors and the dysfunctional agent market, herd mentality. And in Poland, there is a significant relationship between executive pay comparison behaviors and the ineffectiveness of government intervention, herd mentality. The implications of the study are also discussed.

## Introduction

Executive compensation has been rising rapidly worldwide in recent years. Relevant research institutions in the United States have found that in 2016, there were eight listed companies whose CEO compensation exceeded the US $ 30 million^1^ (Market Watch, 2017). The stricter government regulation, the higher executive compensation (Murphy and Jensen, 2018). The compensation of the new Air France president reached 4.25 million EUR^2^ (China Aviation Daily, 2018), which may mark a new peak. Similarly, in China, the “Government Pay Restrained Policy” has been implemented for 3 years, but executive compensation has risen without falling^3^ (CBN, 2019).

The principal–agency theory assumes that agents tend to pursue their interests and that principals, therefore, need to incentivize agents, with pay contracts being one of the primary means of incentivization (Murphy, 1986; Jensen and Meckling, 2019). However, individual perceptions of pay incentives are more social than traditional agency theory (Gartenberg and Wulf, 2017), and agents do not “see things the same way” as their principals. They define their capabilities and values by comparison (Festinger, 1954). Pay is often used as a benchmark for comparison (Kacperczyk and Balachandran, 2018), which may lead to a higher overall pay level.

To a certain extent, an excessive rise in executive compensation can lead to a growing pay gap between executives and ordinary workers or executives, ultimately causing employee discontent, social inequalities, and potential social instability (Gartenberg and Wulf, 2020). Therefore, executive compensation needs to be controlled. The European Countries (Finland, Romania, Germany, Portuguese, and Poland) enacted draft legislation in 2017 to propose new capital requirements, as well as a variety of transparent and open measures to supervise the rapid growth of executive compensation, to stop anomalous bank bonuses, and thus to promote the healthy development of the banking industry and avoid potential financial crises. Most of the larger banks operating in Poland pursued a conservative policy of setting their executive compensation structures (Sturesson, 2015) so that they could select a beneficial structure. The Chinese government has also taken measures to regulate executive compensation. Still, unfortunately, these actions did not achieve the expected results^4^ (China.com, 2017), and recently, “pay restriction orders” have been reissued for state-owned financial enterprises^5^ (China Ministry of Finance, 2022). In the financial industry, there is no specific standard for the compensation of bank executives in China, and many banks, under the guise of marketization, have been increasing their executive compensation, with other enterprises moving closer to them year by year. This shows that enterprises in China (especially in the financial industry) have a severe comparison to executive compensation. Moreover, in a monopoly industry, the evaluation of executives is based on their performance and the use of “global benchmarks” (Cabral et al., 2016; Keller and Olney, 2021) to increase their compensation within a monopoly environment. Therefore, to some extent, executive compensation is not entirely determined by traditionally assessable factors (performance, ownership, etc.). Without specific criteria, executives tend to compare their compensation with peers in the same group and thus increase their compensation (De Vaan et al., 2019).

This paper analyzes the factors influencing executive pay comparison behaviors in China and Poland. The two countries were chosen for comparison for three main reasons: first, Poland is one of the largest and most dynamic economies in the CEE region in terms of recent GDP, population, and interconnectedness with Western Europe (Sahakiants and Festing, 2019). China is the second largest economy in the world, and it is clear that both countries play an important role in their respective regions and the world. Second, Poland was one of the first Communist countries to start the transition from a centrally planned economy to a market economy (Lipton, 1990; Hegarty and Tihanyi, 1999). By the end of 1989, Poland’s centrally planned system collapsed, and the Central and Eastern European economies were all in weak shape. This led to Poland’s transition (Bienkowski, 2002) from a centrally planned economy to a market economy (Brewster and Bennett, 2010), the separation of economy from politics (Kostera, 1995), and the privatization of state enterprise (Baltowski and Mickiewicz, 2000). Both China and Poland are former planned economies and currently emerging market economies. Therefore, they have some historical and external environment comparability. Finally, the Polish market is relatively more open, most banks are under the control of foreigners (Slomka-Golebiowska and Urbanek, 2016), while state-owned enterprises still monopolize many Chinese industries. Thus, a comparison can be made of the degree of marketization of executive pay comparison behaviors in the two countries.

This paper intends to further investigate executive pay comparison behaviors. Data on executives of Polish and Chinese companies will be collected through field research to analyze the differences in executive pay comparison behaviors between the two countries and the factors influencing the pay comparison behaviors of executives in both countries.

### Literature review

Existing studies on executive compensation mainly focus on three aspects: the concept and composition of compensation (Murphy, 1999; Edmans et al., 2017), the comparison of executive compensation across countries and institutional environments (see Herdan and Szczepanska, 2011; Sánchez-Marín et al., 2022), and the factors affecting executive compensation, including performance (Jensen and Murphy, 1990; Elsayed et al., 2018), size (Herdan and Szczepanska, 2011; Iatridis, 2018), internal control (see cook et al., 2019; Lozano-Reina and Sánchez-Marín, 2020), ownership structure (Shan and Walter, 2016; Sánchez-Marín et al., 2022), the institutional environment (Sahakiants and Festing, 2019; Scherer, 2020), and executives’ personal factors (Humphery-Jenner et al., 2016; Conyon et al., 2019).

Scholars have recently begun exploring different influencing factors, such as Confucian culture (Jin et al., 2022). As can be seen, newer research is starting to focus progressively on the impact of culture on executive compensation. Indeed, traditionally, Chinese people believe in “not having a problem with scarcity but having a problem with unevenness.” In reality, some people have a “red eye” and like to compare, especially enjoy comparing themselves with people who are better than them, and scholars have found that employees compare themselves to their managers and peers (Gartenberg and Wulf, 2017; Cullen and Perez-Truglia, 2022), these compare need to work through a medium, a benchmark (De Vaan et al., 2019), and compensation is a benchmark often used for comparison (Obloj and Zeng, 2017; Kacperczyk and Balachandran, 2018).

People will care about their absolute and relative compensation (e.g., Frank, 1984), and social comparison influences pay level and the sensitivity of pay for performance (Gartenberg and Wulf, 2017). In addition, increased competition leads to greater pay for performance sensitivity among the higher-paid managers within firms, while it leads to greater overpayment among others (Gartenberg and Wulf, 2020). Therefore, pay comparison widely exists in executives of a similar status. Generally, people tend to choose better or more professional people than themselves for comparison and to engage in “upward comparisons” (Burnell et al., 2019). If high-income earners’ capital and energy investments are lower than or equal to the executives, the executives will choose passive responses and compare. Executives may adjust their labor by shortening the labor time and reducing labor intensity to “make up” the gap, resulting in low economic efficiency (Li and Liu, 1986; Cullen and Perez-Truglia, 2022). Therefore, the phenomenon of executive pay comparison behaviors should be taken seriously, and the antecedents of executive pay comparison behaviors should be explored in depth to curb executive pay comparison behaviors at the source.

However, most of the studies, data, and information on executive pay comparison behaviors come from the United States (Gartenberg and Wulf, 2017, 2020; Cullen and Perez-Truglia, 2022). In China, scholars explained the apparent phenomenon of executive pay comparison behaviors (Ge and Gao, 2013; Luo et al., 2016; Luo and Zeng, 2018) and the negative impact (Luo et al., 2016; Luo and Zeng, 2018). Research on executive compensation in Poland is sparse. Using “executive compensation” as a keyword, Google returns approximately 2,200,000 results. When using “Poland executive compensation” as a search keyword, only about ten thousand results were acquired, Among the results obtained, Poland is often a part of the CEE countries, and an example that explains one phenomenon, occupying a small space, and the literature on the specialized study of executive compensation is <10, and there are no studies on executive pay comparison behaviors in Poland. Therefore, it is of some theoretical and practical importance to explore executive pay comparison behaviors and factors influencing executive pay comparison behaviors in both China and Poland.

To explore executive pay comparison behaviors and factors to affect executive pay comparison behaviors, this study introduces social comparison theory (Festinger, 1954), and social information processing theory (Salancik and Pfeffer, 1978). This study suggests that executive comparison behaviors will influence executive compensation based on social comparison theory and social information processing theory. According to social information processing theory, individuals, as adaptive organisms, adapt attitudes, behavior, and beliefs to their social context. This leads to the situation that one can learn most about individual behavior by studying the informational and social environment within which that behavior occurs and to which it adapts (Salancik and Pfeffer, 1978: 226). Whereas according to social comparison theory, Festinger (1954) offered some insights into processes of informational social influence in his theory of social comparison. Social comparison is a deliberate act of the individual, an individual always uses similar others for comparison (Festinger, 1954), and compensation is the benchmark often used for comparison (Kacperczyk and Balachandran, 2018). Therefore, individuals make social comparisons based on information about individuals’ compensation, and the greater the individual herd mentality, the more serious the pay comparison. Additionally, the social environment in which people live provides information that influences their attitudes and behaviors. People process the social information around them to better understand their work environment, and in turn, this information-processing process shapes their subsequent attitudes and behaviors (Salancik and Pfeffer, 1978). Agent market, monopoly, and ineffectiveness government intervention belong to the social environment. Accordingly, Executives will understand their situation through these surrounding social environments, and this information process will shape their subsequent attitudes and behaviors (executive pay comparison behaviors).

### Dysfunctional agent market and executive pay comparison behaviors

Social information processing theory proposes that organizations use the market to evaluate personnel, force employees to market-test their worth, can develop a less friendly disposition towards the organization and their work. Some individuals may avoid looking at the external market because if they realize by comparison that there are alternatives in the market, it will make them less satisfied with the current situation and, in turn, affect their work attitudes and behavior (Salancik and Pfeffer, 1978). Thus, markets can impact individuals’ work attitudes and behaviors.

As the complexity of enterprises has increased, the demand for management capacity has also increased, especially in family businesses. Many successors are the family’s offspring (Schlömer-Laufen and Rauch, 2020). Sometimes, a family may want to appoint non-family managers to take over the business when their offspring is low on idiosyncrasy (Zaudtke and Ammerman, 1997). However, some families also reduce the appropriate risk of engaging nonfamily managers by handing over their business to long-serving employees who have proven their trustworthiness (Lee et al., 2003).

Similar to studies on incentive contracting based on the principal-agency theory (Garen, 1994; Sánchez-Marín et al., 2022), Lee et al. (2003) state that, based on transaction costs, an incompleteness of contracts and the hazards of opportunism in engaging agents have also been found. There are competing labor markets with outside agents available to take over the business. Therefore, competitive compensation exists for the agent. Once they are recruited, the competitive labor situation will transform into one of small numbers of bargaining (Williamson, 1979) between the agent and the family business. With coordination costs and risks continuing to rise, as well as their personal preferences, companies tend to train and select the “puppet” type of professional managers because they can use this strategy to decrease the risks that an excellent manager may take but also to retain absolute power. Moreover, the consensus has been that misaligned incentives, a lack of transparency, and moral hazards caused by implicit guarantees have recently led to market failures in the broader environment of the financial markets (Min, 2014). Stimulated by this environment, agents often choose to start their own businesses; the executives’ long-term accumulation of social resources (connections to people and capital) also enhances their ability to do that (Li, 2003). This has resulted in a reduced supply of professional managers, meaning that the agent supply does not equal the demand in this market. So agent compensation depends not on a normal market but on a dysfunctional market.

Jensen et al. (2004) indicate that most company compensation relies on “external market standards” to determine the structure and level of executive compensation. When a manager’s supply decreases, this raises the standard of compensation provides motivation to increase the level of executive compensation, and prompts executive compensation to be close to the benchmark. Managers compare themselves to others whose compensation is higher than theirs, thus increasing their compensation. Take the banking industry as an example in Poland, all banks are controlled by a single, easily-identifiable, large shareholder, and most are under foreigners’ control, so local executives are supervised by foreigners (Slomka-Golebiowska and Urbanek, 2016). This makes some executives feel the owner lacks trust, so they cannot work wholeheartedly. While in China, there is a lack of a manager market (Chen and Fang, 2020). Although most companies expect to hire professional managers (Li, 2003), due to the deep-rooted influence of Chinese traditional culture, usually the offspring are appointed to be the managers (Schlömer-Laufen and Rauch, 2020). Experienced managers will only be recommended when the offspring’s are unwilling to inherit their father’s career. But there may be distrust between the professional managers and the owners. The problem of the principal-agent relationship still exists. To some extent, the Chinese manager market is malfunctioning. Thus, we propose the following hypothesis:

*H1*: Dysfunctional agent market will be positively related to executive pay comparison behaviors.

### Monopoly and executive pay comparison behaviors

Salancik and Pfeffer (1978) stated that satisfaction with the intrinsic aspects of the job decreases when external pay and benefits are more prominent. In other words, when individuals perceive that executives in the same industry are better paid than they are, their attitudes and behaviors change.

Some scholars (Frydman and Saks, 2010; Sabanci and Elvira, 2020) have found that executive compensation varies greatly among different industries and that industry characteristics significantly affect the level and structure of executive compensation. In industries with a high degree of monopoly, corporate executives can apply pressure more easily on the board of directors to develop a compensation contract that will benefit them through internal control (Main et al., 1995). Meanwhile, executives have a relatively larger power than others in many countries, and thus they can use this to control the behaviors of the boards of directors (Bebchuk et al., 2002). In China, this is obvious in monopolized industries, which are the “status industries” (tobacco, petroleum, and petrochemical). In Poland, the reform of the economy retained control over the goods and services field, such as public utilities, public transportation, and goods produced in highly concentrated sectors, such as coal, where monopolistic practices might be expected (Lipton, 1990), in addition to the fact that all banks are controlled by a single, easily identifiable, large shareholder (Slomka-Golebiowska and Urbanek, 2016). As the degree of monopoly increases, an executive’s power will be much larger, and executives can self-price their compensation in this environment; with contractions in benefits, they will compare their compensation to other higher benchmarks. The executive compensation baseline is also raised, and corporate executives will compare their compensation with this baseline. This benchmark compensation concept can also explain the dramatic growth of US corporate executive compensation (Pittinsky and DiPrete, 2013) and the reasons for the outrageous executive compensation in China and some European countries. Therefore, we propose the following hypothesis:

*H2*: Monopoly will be positively related to executive pay comparison behaviors.

### Ineffectiveness government intervention and executive pay comparison behaviors

Salancik and Pfeffer (1978) argued that some literature on the salience of information and field studies of intrinsic and extrinsic reasons support their arguments. That is, one’s own behavioral choices are attributed to extrinsic reasons, such as government intervention.

Mantzavinos stated (2001:11), “the exploration of individual behavior within its social setting, human behavior is influenced by culture and institutions.” As such, human actions are often influenced by institutions. Therefore, it is necessary to consider the social environment, especially institutions and government, when analyzing the behaviors of individuals (Mantzavinos, 2001). To prevent executives from abusing their power and increasing their compensation, corporate shareholders have taken many measures, such as introducing strategic investors, establishing a remuneration committee, hiring outside directors, etc., (Conyon, 2006; Kanapathippillai et al., 2019). However, company power is still highly concentrated in the hands of the executives. Moreover, the power of corporate executives and the personal benefits they can obtain are positively correlated. With growing power, executives will get used to manipulating earnings to enhance performance-related pay (Basu et al., 2007). In this case, the market (invisible hand) fails to adjust compensation to the position, and the only party that can limit and control executive pay is the government. The government can use the “visible hand” (legal, economic, and administrative measures) to intervene. However, in China, the government has not effectively regulated executive compensation among listed companies (Huang and Xi, 2009; Chen et al., 2010). This phenomenon also exists in the United States and European countries (Murphy and Jensen, 2018), as government workers always use their power to “free ride” (benefit from other people’s efforts). Given the ineffectiveness of the government, executives in state-owned companies with no control will compare themselves to other people whose higher compensation is then increased. In Poland, before 2012, there were no specific laws on the level and structure of executive compensation in the financial sector institutions. The only issue subject to regulation was the transparency of executive compensation in listed companies (Slomka-Golebiowska and Urbanek, 2016), while many listed companies in Poland do not publish information on directors’ remuneration (Herdan and Szczepanska, 2011). So the government intervention was ineffective. In China, the government has been attaching great importance to executive compensation chaos and adopted a “Pay Restrained Policy” to regulate the compensation of executives. However, the expected results were not achieved. The payment of corporate executives remained high and even continued to rise (the annual report of listed companies in 2016 shows that behind the “Pay Restrained Policy,” the chairman of many listed state-owned enterprises no longer receives compensation from listed companies, while the average annual compensation of a state-owned enterprise chairman who is still receiving compensation shows an upward trend, so to some extent, the intervention of Chinese government is also ineffective. Thus, we propose the following assumption:

*H3*: The ineffectiveness of government intervention will be positively related to executive pay comparison behaviors.

### Herd mentality and executive pay comparison behaviors

According to social comparison theory (Festinger, 1954), in the absence of objective criteria, each individual uses others as a yardstick for comparison to self-evaluate. Specifically, firstly, people are driven to evaluate their own opinions and abilities; secondly, non-social means are to some extent inapplicable, so people evaluate their own opinions and abilities by comparing themselves with others; finally, individuals choose to compare themselves to others who are similar to them, and these comparisons are an important cause of their psychological change.

Herd mentality is an alignment of individuals’ thoughts and/or behaviors in a group that emerges without purposeful coordination by a central authority or leading figure and instead through local interactions among agents (Kameda and Hastie, 2015; Loxton et al., 2020). The herd mentality can cause irrational psychology and behaviors (Loxton et al., 2020). With an inefficient market, the market cannot naturally regulate. When some corporate executives earn high compensation, others will be psychologically imbalanced, and they will not take experience, competence, and performance into consideration, thus leading to blind comparisons: first, they will compare to developed countries; second, they will compare to similar companies; third, they will compare to other companies in their region (Liu et al., 2014). This phenomenon is particularly evident in state-owned enterprises because the market does not select executives in state-owned enterprises but appointed by the government directly (Xinhua, 2018)^6^, and this selection process may also lead to many problems. They enjoy national privileges and benefits (from both the government and the company), so they may use information about government people unsuitable for a company. Executives at the same level and industry pursue the same treatment and compensation. When corporate executives begin to make blind comparisons because of the herd mentality or because they have a sense of comparison, this will make the executive pay generally higher than the market equilibrium and thus lead to the so-called astronomical rises in compensation. Since herd mentality is a psychology that affects everyone, individuals communicate with others in their environment once it happens. These acts of communication lead to shared mental models, which may result in executive pay comparison. Herd mentality is the nature of humans, and social comparisons are ubiquitous (Campbell et al., 2017), so it is the same in China and Poland. Therefore, we propose the following hypothesis:

*H4*: The herd mentality will be positively related to executive pay comparison behaviors.

## Materials and methods

### Procedure and participants

The sample for this study is drawn from China and Poland, including EMBA students in universities and corporate executives (an executive is defined as a company executive manager, assistant manager, chief financial officer, secretary of the board of directors of listed companies or other equivalent position), the research method was mainly through face-to-face guidance, while a small amount was by mail. We designed a preliminary scale questionnaire based on existing literature and expert opinions to verify the above assumptions. First, we selected 30 persons to do the pretest (these results were not included in the final survey sample) in China and Poland (collected mainly in Poland, while Eastern European countries all conclude). During the formal research phase, we distributed 260 questionnaires in China, the final number of valid responses was 161, corresponding to an effective rate of 61.92%. A total of 237 questionnaires were distributed in Poland, the final number of valid responses was 118, most of the uncollected questionnaires were distributed by email, and the effective rate was 49.79%.

### Reliability and validity analysis

Based on the pretest feedback, SPSS24.0 was used for conducting the reliability and validity analysis. The results are as follows: in China, the Cronbach’s coefficients of each of the factors (Cronbach’s) are close to 0.8, with an average of 0.812, which indicates that the entire scale is well reliable. In Poland, the Cronbach’s coefficients of each of the factors (Cronbach’s) are close to 0.8, with an average of 0.817, indicating that the full scale is well reliable. In China, we did the KMO and Bartlett test of sphericity value analysis for the executive pay comparison behaviors factors scale. The results showed that the KMO value (=0.702) was greater than 0.7 and that the Bartlett (Bartlett) test of sphericity was significant (*p* = 0.000). We then extracted two factors whose characteristic values are greater than one: monopoly and herd mentality. Their cumulative variance explained rate was 65.97%. This indicates that the validity of the entire scale is good. In Poland, we did the KMO and the Bartlett test of sphericity value analysis for the executive pay comparison behaviors factors scale. The results show that the KMO value (=0.714) is greater than 0.7 and that the Bartlett (Bartlett) test of sphericity was significant (*p* = 0.000). We then extracted two factors whose characteristic values are greater than one: the dysfunctional agent market and herd mentality, whose cumulative variance explained rate was 76.389%. This indicates that the validity of the entire scale is good. The last revision was made to the questionnaire to make it more in line with the expression habits of the executives so that they could find it easier to understand and fill it in.

### Measures

To ensure the accuracy of the empirical analysis, this research scale refers to authoritative journal literature. Three PhDs in the field were invited to translate and back-translate the scales, and two experts in the field were invited to investigate the scales and adapt them as appropriate. Five-point Likert scale was used to measure all variables.

#### Pay comparison behaviors

The eight items were set based on Festinger (1954) and Li and Liu (1986). Example items are, “Found your income returns are lower than your peers or colleagues, you would choose to jump ship to other companies willing to give you a higher return.” The Cronbach’s α for this scale was 0.809 (China) and 0.784 (Poland).

#### Dysfunctional agent market

The three items were set mainly based on Li (2003) view on the mismatch between the demand and supply of managers. Typical statements in the scale include “For your company’s executives, the situations in which jobs can not match with personal competence appears.” The Cronbach’s α for this scale was 0.814 (China) and 0.821 (Poland).

#### Monopoly

The three items were set based on the expressions of market monopoly manifested in the Anti-Unfair Competition Law. Typical statements in the scale include “in your opinion, the situation of some natural monopoly (water, electricity, gas, etc.) and having monopolistic status’ industries’ (tobacco, petroleum, petrochemical) monopolistic behavior (forced transaction, overcharging) of your company’s industries is?” The Cronbach’s α for this scale was 0.850 (China) and 0.799 (Poland).

#### Government intervention

The three items were set based on three primary forms of government intervention. Specific questions are developed to describe the phenomenon by legal, economic, and administrative means. Typical statements in the scale include “The effect which government takes legal and economic instruments (such as policy development, adjusting tax rates, subsidies, etc.) on your company is?” The Cronbach’s *α* for this scale was 0.803 (China) and 0.831 (Poland).

#### Herd mentality

The four items were set based on Corneoa and Jeanne (1997) analysis of the manifestations of herding. Specific questions are established in various ways and from several domains to measure whether corporate executives “follow the herd.” Typical statements in the scale include “If many people are buying one thing, you will go to buy.” The Cronbach’s *α* for this scale was 0.782 (China) and 0.850 (Poland).

### Control variables

Because individual differences in experience and professional affiliation may affect an employees’ attitudes toward his or her work (Bunderson and Thompson, 2009), we controlled for the following variables: age (1 = 20–30; 2 = 31–40; 3 = 41–50; 4 = more than 50) and tenure (1 = less than 1 year; 2 = 1–3 years; 3 = 3–5 years; 4 = 5–8 years; 5 = more than 8 years).

### Basic statistical characteristics

In this paper, we conduct a comparative statistical analysis (Table 1) between China and Poland of the basic characteristics in the sample questionnaire, and the results are as follows:

**Table 1:** Basic characteristics of the sample.

	Items	Sample distribution					Total (%)
China	Comparison Targets	Abroad	Domestic	Within the company	Other	Do not compare	
		11.8%	72.7%	5%	2.5%	8.1%	100
	Age	20–30	31–40	41–50	More than 50		
		6.8%	52.2%	38.5%	2.5%		100
	Duration	Below 1 year	1–3	3–5	5–8	More than 8	
		2%	17.4%	25.5%	14.9%	41%	100
Poland	Comparison Targets	Abroad	Domestic	The company	Other	Do not compare	
		9.1%	54.5%	10.9%	5.45%	20%	100
	Age	20–30	31–40	41–50	More than 50		
		27.3%	61.8%	10.9%	0.0%		100
	Duration	Below 1 year	1–3	3–5	5–8	More than 8 years	
		10.9%	23.6%	23.6%	20%	21.8%	100

First, after comparing the primary data between China and Poland, we can conclude that the two countries are similar, with most corporate executives from both countries regarding domestic counterparts as pay comparison objects, accounting for 72.7 and 54.5%, respectively. However, it is worth mentioning that the number of respondents who do not compare their pay with others is larger in Poland than in China, accounting for 20 and 8.1%, respectively, indicating that executive pay comparison behaviors are more prevalent in China. Second, regarding the age of executives, Polish executives are younger compared to China. No Polish respondents were older than 50 years, which is different from China. Finally, regarding the years of executives’ service, Polish executives have served for a shorter term, while Chinese executives have mostly served more than 5 years. On the one hand, this indicates that Chinese executives may be more loyal than their Polish counterparts; on the other hand, this may also mean that it takes Chinese employees longer to reach an executive position.

## Statistical results

### Correlation analysis

Using SPSS24.0 software to conduct the Pearson correlation analysis, we obtain the following findings: in China (Table 2), executive pay comparison behaviors positively correlate with herd mentality (*r* = 0.185, *p* < 0.05); however, there is no relationship between executive pay comparison behaviors and ineffectiveness government intervention, monopoly, or herd mentality. In Poland (Table 2), executive pay comparison and monopoly are positively correlated (*r* = 0.332, *p* < 0.05), and it is also significantly positively correlated with herd mentality (*r* = 0.297, *p* < 0.05). At the same time, there is no correlation between executive pay comparison behaviors and other influencing factors.

**Table 2:** Correlation analysis result between the relevant variables.

	Variable	*M*	*SD*	1	2	3	4	5	6	7
China	1. Executive pay comparison behaviors	17.155	2.919	1	−0.151	−0.142	0.021	0.185*	0.155	0.082
	2. Dysfunctional agent market	9.335	2.318	−0.151	1	0.173	−0.013	0.053	0.33	−0.091
	3. Monopoly	7.491	1.647	−0.142	0.173	1	0.060	−0.026	0.13	−0.053
	4. Ineffectiveness of government intervention	9.665	2.670	0.021	−0.013	0.060	1	0.027	−0.099	0.31
	5. Herd mentality	11.360	2.002	0.185*	0.053	−0.026	0.027	1	0.074	−0.073
	6. Age	2.367	1.237	0.155	0.13	−0.099	0.074	−0.22	1	0.086
	7. Tenure	3.789	0.649	0.082	−0.091	−0.053	0.13	−0.073	0.086	1
Poland	1. Executive pay comparison behaviors	21.582	3.457	1	0.219	0.033	0.332*	0.297*	0.136	0.435
	2. Dysfunctional agent market	8.382	2.805	0.219	1	0.023	0.109	−0.211	0.084	0.311
	3. Monopoly	11.636	2.460	0.033	0.023	1	0.073	−0.211	0.234	0.087
	4. Ineffectiveness of government intervention	10.146	2.013	0.332**	0.109	0.073	1	0.340	−0.149	0.111
	5. Herd mentality	11.346	2.977	0.297**	−0.211	−0.211	0.340	1	−0.185	−0.214
	6. Age	1.836	0.601	0.136	0.084	0.234	−0.149	−0.185	1	0.738
	7. Tenure	3.182	1.321	0.435	0.311	0.087	0.111	−0.214	0.738	1

*p < 0.05, **p < 0.01.

### Regression analysis

We used SPSS 24.0 to do a linear regression analysis to reveal the relationship between various factors and executive comparative pay, and we obtained the following results for China (Table 3). First, there is a significant relationship between executive pay comparison behaviors and dysfunctional agent market, herd mentality. At the same time, there is no relationship between executive pay comparison behaviors and the other two factors.

**Table 3:** The relationship between executive pay comparison behaviors and various affecting factors: OLS regression results.

	Executive pay comparison behaviors (China)		Executive pay comparison behaviors (Poland)	
Ineffectiveness of government intervention	Sig	0.923	Sig	0.013
	Number of samples	161	Number of samples	118
Dysfunctional agent market	Sig	0.048	Sig	0.552
	Number of samples	161	Number of samples	118
Monopoly	Sig	0.216	Sig	0.638
	Number of samples	161	Number of samples	118
Herd mentality	Sig	0.018	Sig	0.028
	Number of samples	161	Number of samples	118
Age	Sig	0.074	Sig	0.263
	Number of samples	161	Number of samples	118
Tenure	Sig	0.694	Sig	0.272
	Number of samples	161	Number of samples	118

The same linear regression analysis as in China was conducted for the Polish sample (Table 3). We obtained the following results: there is a significant relationship between executive pay comparison behaviors and ineffectiveness of government intervention, herd mentality. There is no relationship between executive pay comparison behaviors and the other two factors.

## Discussion

Through comparative research of the two countries, the following conclusions can be reached:

First, executive pay comparison behaviors exist in both China and Poland, but the number of respondents who do not compare their pay with others is higher in Poland than in China. Second, the dysfunctional manager agent market is one reason for executive pay comparison behaviors in China, whereas this is not apparent in Poland. China has a strong family culture but lacks a stable external labor market (Kim and Gao, 2013). Indeed, Li (2003) studied family firms in economically developed areas of eastern coastal China and found that agency market failures were widespread. However, in today’s complex economy and highly competitive society, when the agent’s market is dysfunctional, professional managers face greater market competition outside the organization and less room for career development within the organization, they will focus more on their career development than on pay comparison. Thus, the results show a negative relationship between the dysfunctional agent market and executive pay comparison behaviors. Third, herd mentality is a common factor that causes executive pay comparison behaviors in the two countries. Herd literature suggests that people tend to discount their beliefs and imitate others when making adoption decisions (Sun, 2013), and the herd mentality can result in anxiety and irrational behaviors (Sherman et al., 2021). So it is not difficult to explain that herd mentality is the same factor that causes executive pay comparison behaviors in the two countries. Fourth, ineffective government intervention is one factor that causes executive pay comparison behaviors in Poland, whereas this is not apparent in China.

In summary, the market is the main reason for the differences between the two countries. China and Poland used to be planned economies and are now emerging market economies. While they may face similar situations, their different degrees of marketization can also lead to different outcomes.

### Theoretical contributions

This study makes the following theoretical contributions: (1) The introduction of executive comparison into examining executive compensation issues has enriched and improved the theoretical analysis of executive compensation. Previous research on the influence of executives’ personal effects on compensation has focused on the influence of executives’ wealth (Becke, 2006) and personal experience (Conyon et al., 2019), ignoring the influence of executives’ psychological effects on compensation. Additionally, recent research has found that employees compare themselves to their managers and peers (Gartenberg and Wulf, 2017; Cullen and Perez-Truglia, 2022). According to social comparison theory, People evaluate themselves by comparing themselves with others (Festinger, 1954), and tend to choose people who are better or more professional than themselves to engage in “upward comparisons” (Burnell et al., 2019). The executive pay comparison behaviors discussed in this study, which is a comparison between executives and individuals of the same person in the unit, complements existing research. (2) The questionnaire survey from two countries (China and Poland) are significant in revealing executive pay comparison behaviors. While existing domestic and international studies on executive pay comparison behaviors are almost always focused on individual countries (Luo et al., 2016; Luo and Zeng, 2018; Gartenberg and Wulf, 2020). This study compares executive pay comparison behaviors in Poland and China, which is the first attempt to make a cross-cultural comparison, advancing the study of multicultural contexts in this field. (3) The first time that the factors influencing executive pay comparison behaviors are analyzed and explored. Existing research on executive pay comparison behaviors mainly encompasses the impact of comparison behaviors on compensation (Liu et al., 2014; Gartenberg and Wulf, 2017) and an exploration of the consequences of pay comparison (Luo et al., 2016; Luo and Zeng, 2018; Cullen and Perez-Truglia, 2022), this study focuses on the antecedents of executive pay comparison behaviors, making the study of executive pay comparison behaviors more complete.

### Practical implications

In recent years, the management of executive compensation in many state-owned enterprises and listed companies, whether in China or Poland, has seriously gone out of control and excessive compensation has occurred (Liu et al., 2014; Słomka-Gołębiowska, 2016), largely due to the existence of a blind comparison in executive compensation and the lack of effective regulatory countermeasures by the relevant state departments. Curbing the blind rise in executive compensation and maintaining the fairness and stability of the executive compensation market is a pressing issue in the current social economy. The findings of this paper provide new ideas to address this issue.

First, we must eliminate herd mentality to eliminate executive pay comparison behaviors from their roots. This study found that herd mentality positively correlates with executive pay comparison behaviors in China and Poland. That is to say, the stronger the herd mentality, the more executive pay comparison behaviors the executives will get. Asch (1956) confirmed that the main reason for the emergence of herd mentality is mainly due to two aspects: to make most people believe they are initially driven by the desire to be correct and to make a good impression on others. The process of herd mentality that is produced mainly includes three steps: compliance (the start of herd mentality), identity (an individual voluntarily accepts the views, information, or group norms that are consistent with others), and internalization (the final stage of herd mentality). A person may herd depending on the type of person and the group members; generally, people who lack self-confidence and have higher requirements are more accessible to herd. While the higher prestige of group members allows individuals to find a sense of belonging, it can also increase the possibility of individual herding. Therefore, we should restrain the herd mentality at the beginning. In terms of individuals, managers of corporate executives should have some understanding of the character of the executives so that they can take measures to weaken their tendency to make negative comparisons. The formation of small groups used for negative comparisons should be controlled in terms of groups. In terms of the environment, we should cultivate moral rules that are characterized primarily by the fact that they require a kind of behavior that is contrary to the interests of individuals. Typical examples of such ethical rules are “keep promises,” “do not cheat,” “respect other people’s property,” and “tell the truth.” (Mantzavinos, 2001). We should also celebrate high values in the whole society, promote maverick types of personalities, scorn and combat behavior called “going with the flow.” Second, to increase government intervention, the government should play a role that is not just empty. This paper shows a positive correlation between executive pay comparison behaviors and the ineffectiveness of government intervention in Poland, while it is not apparent in China. The function that government intervention can play cannot be ignored: market failures need the “visible hand” of government intervention to supervise companies’ behavior in a political, economic, and legal way. Murphy and Jensen (2018) argue that the reality is that executive pay is already heavily regulated but that these regulations have had little effect. Part of the problem is that regulation is inherently focused on a relatively narrow aspect of compensation, leaving companies plenty of scopes to circumvent regulation by changing other, less regulated parts of their compensation. Therefore, Interventions should strengthen the general supervision and discipline of executives. Externally, it should mainly enhance the supervision of the public, media, and public opinion; internally, it should provide top-down vertical and horizontal restraints among peers for corporate executives.

### Limitations and future research

Although our study has several strengths, such as using two countries for comparative analysis and collecting data in three languages to avoid possible linguistic misunderstandings, our study still has several limitations that should be acknowledged. Firstly, executive pay comparison behaviors are a unique management issue. There is not yet sufficient authoritative literature worldwide to draw on, nor are there established scales that can be used directly. Although we have developed scales concerning the authoritative literature, the results are slightly less representative due to the difficulty of obtaining a large sample of executives. We encourage future research to expand the sample as much as possible to make the scales more broadly representative. Secondly, we used a questionnaire to collect data to test our hypotheses. However, future research may consider other research methods, including qualitative methods, laboratory studies, and other diverse methods to explore issues related to executive pay comparison behaviors. Third, this study focus on the antecedents of executive pay comparison behaviors, it complements existing studies that focus only on the consequences of executive pay comparison behaviors (Luo et al., 2016; Luo and Zeng, 2018; Cullen and Perez-Truglia, 2022), it does not address the outcomes of executive pay comparison behaviors and falls slightly short in completeness, and we encourage future studies to address a more comprehensive and systematic exploration of “antecedents - executive pay comparison behaviors– consequences.” In addition, It would also be interesting to experiment with executive pay comparison behaviors as a moderating variable.

## Data availability statement

The original contributions presented in the study are included in the article/Supplementary material, and further inquiries can be directed to the corresponding author.

## Ethics statement

Ethical review and approval was not required for the study on human participants in accordance with the local legislation and institutional requirements. The patients/participants provided their written informed consent to participate in this study.

## Author contributions

YL and HL conceived and designed the work, YL collected, analyzed, interpreted the data, and drafted the article. YL and HL are responsible for the modifications. All authors contributed to the article and approved the submitted version.

## Conflict of interest

The author declares that the research was conducted in the absence of any commercial or financial relationships that could be construed as a potential conflict of interest.

## Publisher’s note

All claims expressed in this article are solely those of the authors and do not necessarily represent those of their affiliated organizations, or those of the publisher, the editors and the reviewers. Any product that may be evaluated in this article, or claim that may be made by its manufacturer, is not guaranteed or endorsed by the publisher.

## Footnotes

^1^ http://www.marketwatch.com/story/ceo-average-pay-climbed-more-than-1-million-in-2016-2017-04-12

^2^ http://www.chinaaviationdaily.com/news/70/70183.html

^3^ https://www.yicai.com/news/100156198.html

^4^ https://news.china.com/domesticgd/10000159/20170417/30426345_1.html

^5^ mof.gov.cn

^6^ https://baijiahao.baidu.com/s?id=1611864999401186808&wfr=spider&for=pc
